# P-2273. Latent Tuberculosis and Strongyloidiasis Screening in Patients with Hematologic Malignancy Prior to Cytotoxic Therapy in Boston, Massachusetts

**DOI:** 10.1093/ofid/ofae631.2426

**Published:** 2025-01-29

**Authors:** Katherine A Reifler, Taylor Francoeur Smith, Maura A Fagan, Daniel Bourque, J Mark Sloan

**Affiliations:** Boston University School of Medicine, Boston, Massachusetts; Boston University School of Medicine, Boston, Massachusetts; Boston University School of Medicine, Boston, Massachusetts; Boston Medical Center / Boston University School of Medicine, Boston, Massachusetts; Boston University School of Medicine, Boston, Massachusetts

## Abstract

**Background:**

Latent tuberculosis infection (LTBI) affects roughly one quarter of the world’s population and *Strongyloides stercoralis* (SS) affects approximately 100 million people worldwide. Individuals with hematologic malignancy receiving chemotherapy and systemic steroids are at increased risk of reactivation of LTBI and SS dissemination, which are associated with significant morbidity and mortality. However, LTBI and SS screening are not uniformly performed.

**Methods:**

We conducted a quality improvement intervention of universal screening for LTBI and SS for one year (March 2023-March 2024) amongst patients initiating chemotherapy in the hematology clinic at Boston Medical Center, an urban academic safety net hospital with a large immigrant patient population. Screening was encouraged via integration into electronic medical record chemotherapy care plans and by lectures with hematology providers.

**Results:**

876 pre-intervention and 83 post-intervention patients were included. Mean age was 61-63 years (pre- vs post-intervention groups). Plasma cell malignancy was most common (∼46%) followed by B-cell lymphoma (22-32%). Over 90% of individuals received systemic steroids, 2-3% were HIV+, and 2% were HTLV-1+. Between 33-40% of individuals immigrated from countries with high TB incidence rates > 40 per 100,000 people and SS prevalence >10%. The intervention increased LTBI and SS screening rates [TB: 337/876 (38%) pre- vs 49/83 (59%) post-intervention, SS: 159/876 (18%) pre- vs 49/83 (59%) post-intervention], but universal screening was not achieved. Among those screened for LTBI, 73/337 (22%) pre- versus 9/83 (18%) post-intervention were positive. Among those screened for SS, 4/159 (3%) pre- versus 2/49 (4%) post-intervention were positive.

**Conclusion:**

These preliminary findings demonstrate a patient population with risk factors for reactivation and/or dissemination of LTBI and SS. Enhancing screening rates for LTBI and SS may prevent serious complications of these infections and there is a need to improve screening guidelines in these high-risk individuals.

**Disclosures:**

Daniel Bourque, MD, Kephera Diagnostics: Grant/Research Support J. Mark Sloan, MD, Nuvectis pharmaceuticals: Advisor/Consultant

